# Successful Clipping of a Giant Anterior Inferior Cerebellar Artery Aneurysm with Combined Transpetrosal Approach

**DOI:** 10.1155/2019/6049573

**Published:** 2019-09-18

**Authors:** The-Hao Nguyen, Van-Thanh-Cong Pham, Quynh-Trang Pham, Hao Nguyen Si Anh, Phong Tran Nhu, Hoang-Long Vo

**Affiliations:** ^1^Department of Neurosurgery, Bach Mai Hospital, Hanoi, Vietnam; ^2^Institute for Preventive Medicine and Public Health, Hanoi Medical University, Hanoi, Vietnam; ^3^Public Health Department, Nursing Faculty, Dai Nam University, Hanoi, Vietnam

## Abstract

**Background:**

The anterior inferior cerebellar artery (AICA) aneurysms are rare lesions whose treatment can be challenging. There are only a few previous reports of surgical treatment for such lesions.

**Objectives:**

We present a case of a basilar-AICA aneurysm undergoing surgery with the combined transpetrosal approach.

**Case Description:**

A 58-year-old female patient presented clinical signs including headache, diplopia, and right hemiparesis. The radiological imaging showed a basilar-AICA aneurysm measuring 25 × 19 mm. The patient was operated via left combined transpetrosal approach. The outcome was graded mRankin 1. Follow-up computerized tomographic angiography showed no aneurysmal residual and total preservation of basilar artery.

**Discussions:**

Surgical indication's purposes were aneurysmal elimination and reduction of mass effect. Combined transpetrosal approach: proximal segment control and enough space for clipping manipulation. Clipping techniques: Temporary clip for aneurysmal collapsing, “orienting clip”.

**Conclusion:**

Giant basilar-AICA aneurysm is very rare lesion. Combined transpetrosal approach is appropriate for surgical clipping. Good surgical outcome is achieved with complete elimination of aneurysm.

## 1. Introduction

**Figure 1:**
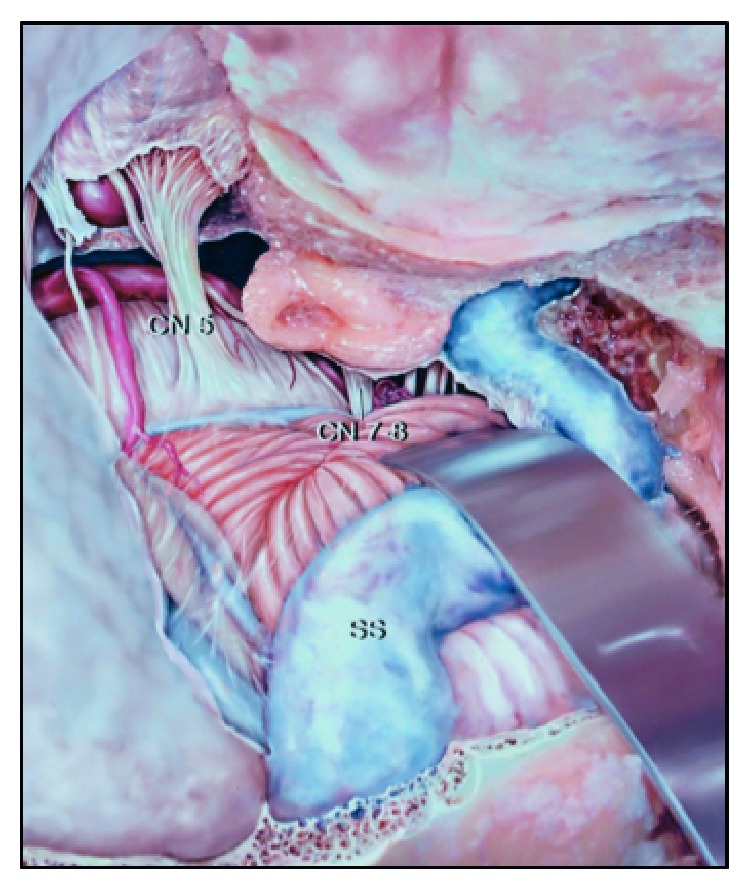
Combined transpetrosal approach schema.

The aneurysms located near the verterbrobasilar junction or the anterior inferior cerebellar artery (AICA) aneurysms are uncommon lesions, accounting for only about 1% of all intracerebral aneurysms. There are important structures in this region concerning middle and lower clivus such as the pontine and the cranial nerves. Surgical approach to this region is still a challenge for neurosurgeons. Samii (1992) named the clivus as “no man's land” [1]. In 1948, Schwartz performed the first surgically treated case of AICA aneurysm clipping [2]. Until 2005, one of the biggest series of AICA aneurysm clipping was reported by Gonzalez with 34 cases. Subtemporal approach described by Drake (1978) was quite successful except for some cases with the vein of Labbé infarct. This approach requires the temporal lobe retraction, but it is still hard to control the lower part of basilar artery [3]. Kawase (1985) reported two cases of AICA aneurysm clipping via anterior petrosectomy without any complications; however, he acknowledged that he had difficulty controlling the proximal artery [4]. In 1996, Kawase reported another series of 14 cases experiencing midbasilar artery aneurysms clipping with anterior petrosectomy, posterior petrosectomy, and far-lateral approach [5]. Lawton (1997) applied modified orbitozygomatic and far-lateral approaches to treat 28 patients with large and giant midbasilar artery aneurysms. Then this surgical treatment outcome was recorded to be 75% patients with good outcome and 11% of those were having permanent treatment-associated neurological deficits [6]. Nowadays, with modern microsurgical instruments and better anatomical understanding, it is easier for neurosurgeons to treat the lesions of this region. The combined transpetrosal approach for the tumoral lesions is quite common; however, it is still controversial for aneurysmal clipping (Figure 1). We present a case of giant AICA aneurysm successfully clipped via combined transpetrosal approach, and evaluate the possibility, the advantages, and the inconveniences of this approach, along with sharing our experiences on used approach.

## 2. Case Presentation

### 2.1. Clinical and Imaging Features

**Figure 2:**
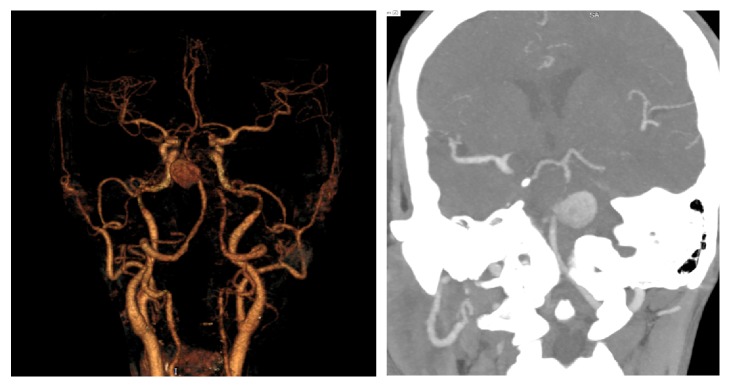
Pre-op CTA.

A 58-year-old woman with medical history of hypertension presented with onset of headache, diplopia, and right sided weakness within 2 weeks. She was admitted to the Department of Neurosurgery (Bach Mai hospital) on 12 February 2018. Here, a cerebral computed tomography (CT) was performed, showing a hyperdense mass in the brainstem. CT angiography (CTA) revealed that a partially thrombosed aneurysm measuring 25 × 19 mm was located at the vertebrobasilar junction (AICA arises), causing mass effect on the pons (Figure 2). The radiologists found that endovascular treatment was a challenge for this aneurysm because of its wide neck and its compression on the brainstem. Our patient underwent the surgery on 28 February 2018 with combined transpetrosal approach.

### 2.2. Operation

**Figure 3:**
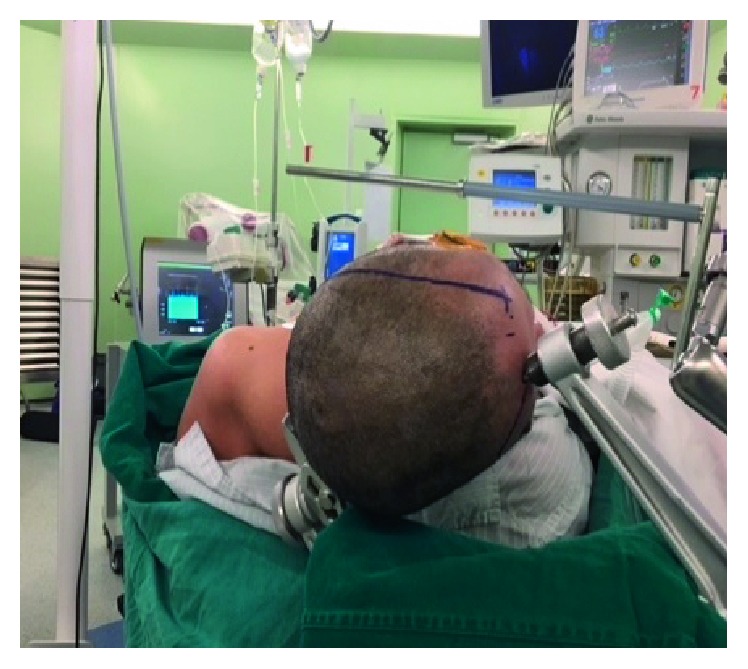
Head position.

**Figure 4:**
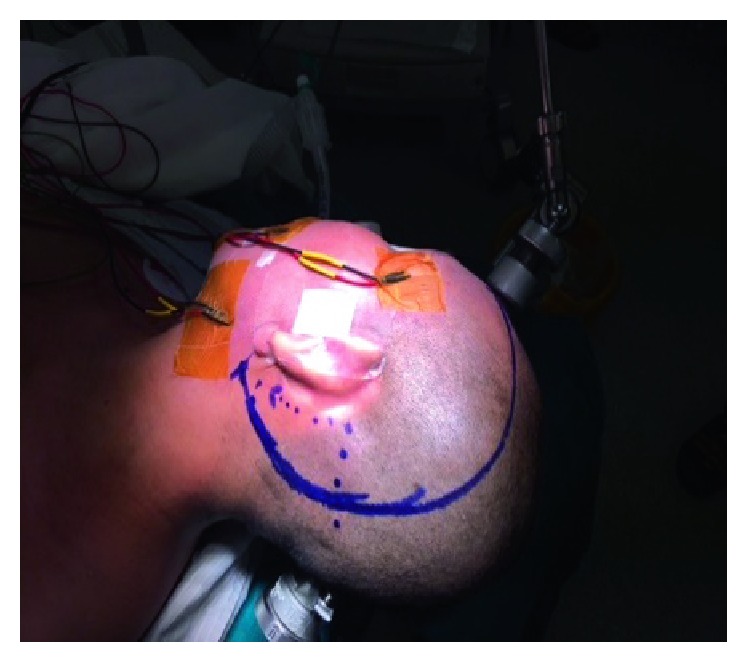
C-shaped incision.

**Figure 5:**
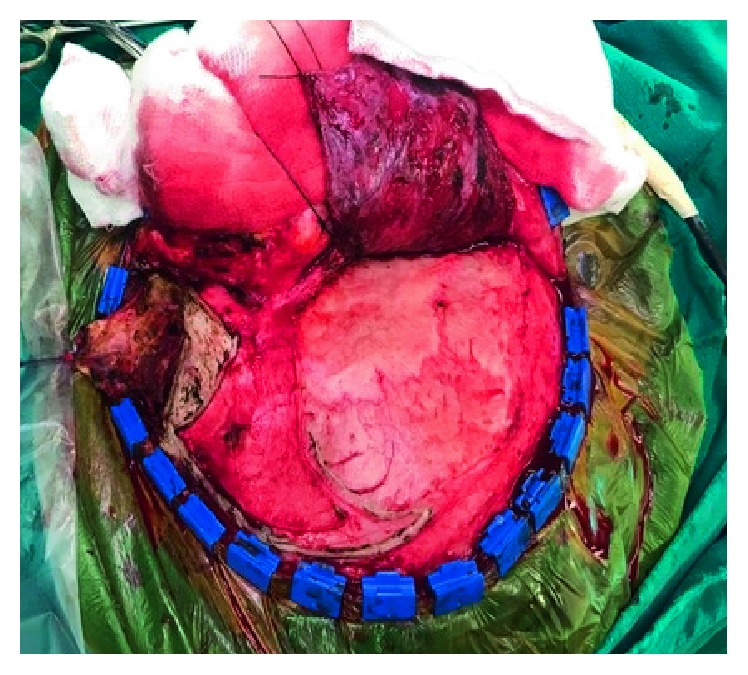
Temporal muscle dissection.

**Figure 6:**
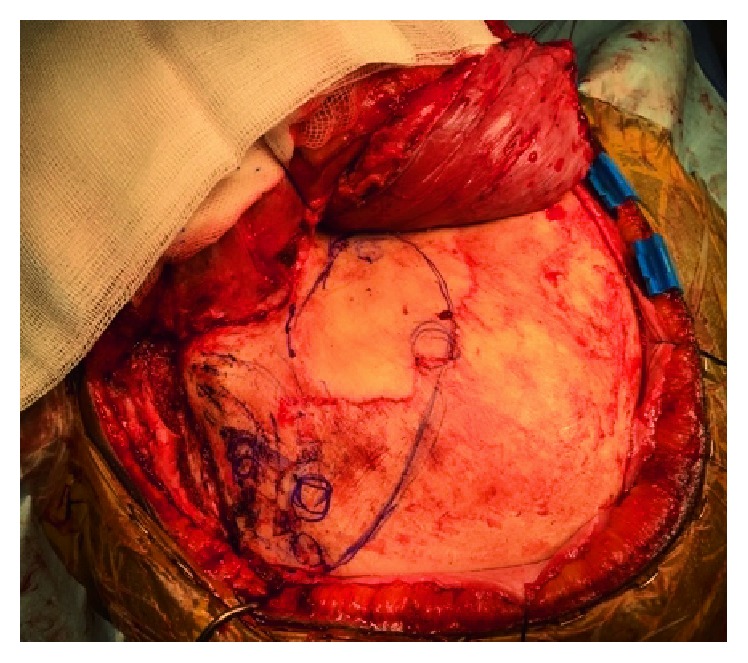
Mastoid drilling.

The patient was in the supine position with the head turned right 80° and extended 30° (Figure 3). C-shaped incision was made from mastoid process to hairline on the left side (Figure 4). Temporal muscle was dissected to expose the temporobasal region as well as zygomatic root, and the lateral suboccipital region as well as the complete mastoid (Figures 5 and 6). The posterior petrosectomy was performed with high speed drill under the microscope to expose the atrium while preserving the semicircular canals, unroofing the sigmoid sinus, and a part of tranverse sinus until the presigmoid dura was exposed. A subtemporal craniotomy was performed for the exposure of Kawase triangle. The anterior part of the petrous bone was totally drilled until the posterior fossa dura and the inferior petrosal sinus were exposed. When the temporobasal dura was opened, the cerebrospinal fluid (CSF) was drained for the brain relaxation. Through the Kawase triangle opening, the posterior fossa dura was incised with two cuts crossing the superior petrosal sinus at anterior one-third and the cerebellar tentorium to its border. Transverse and sigmoid sinuses were retracted posteriorly, then the presigmoid dura was opened close to the inferior border of the superior petrosal sinus. The arachnoid was opened to expose the vertebrobasilar junction, the aneuryms, and the middle part of the basilar artery. One temporary clip was put on the proximal basilar artery through the posterior petrosal opening. The aneurysm wall was torn during the first clip application. Finally, the aneurysm was clipped with two long Yasargil clips through Kawase triangle opening. Dura closure was performed with the free flap of the temporal fascia, the abdominal fat tissue, and the biological glue.

## 3. Results

**Figure 7:**
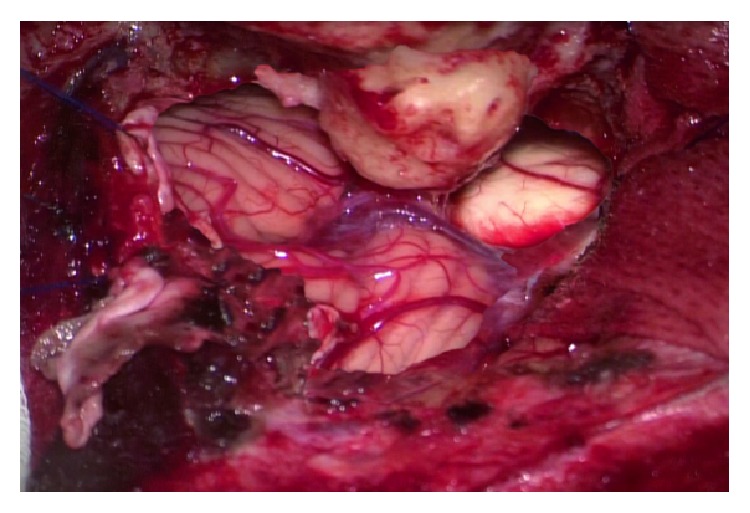
Dural opening.

**Figure 8:**
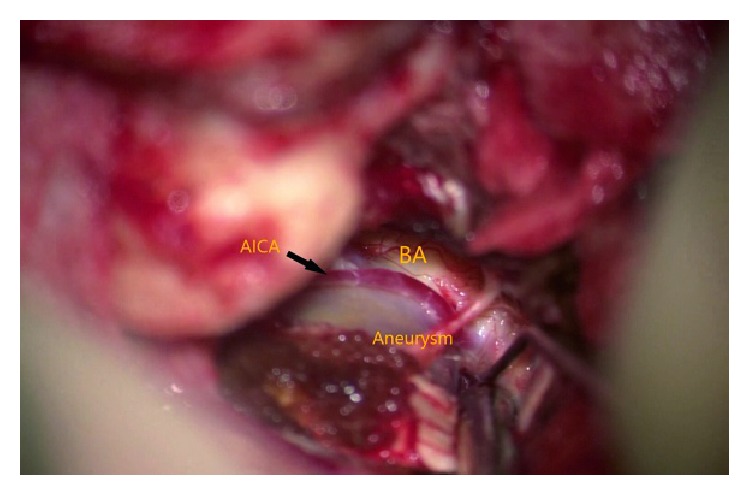
Aneurysm exposure.

**Figure 9:**
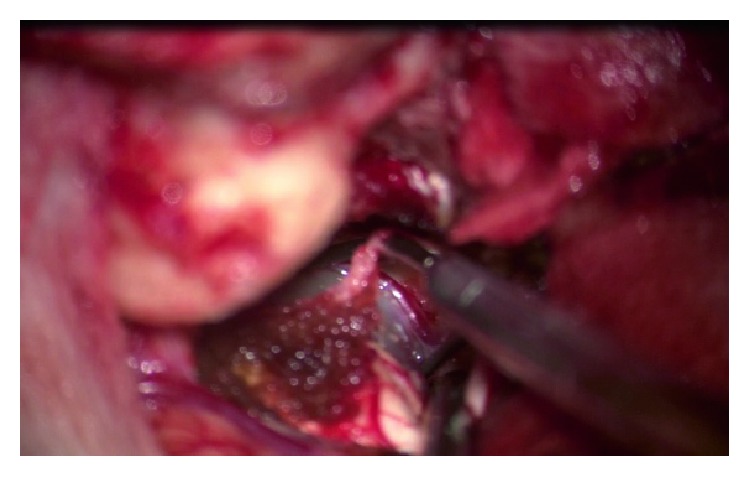
Applying clip.

**Figure 10:**
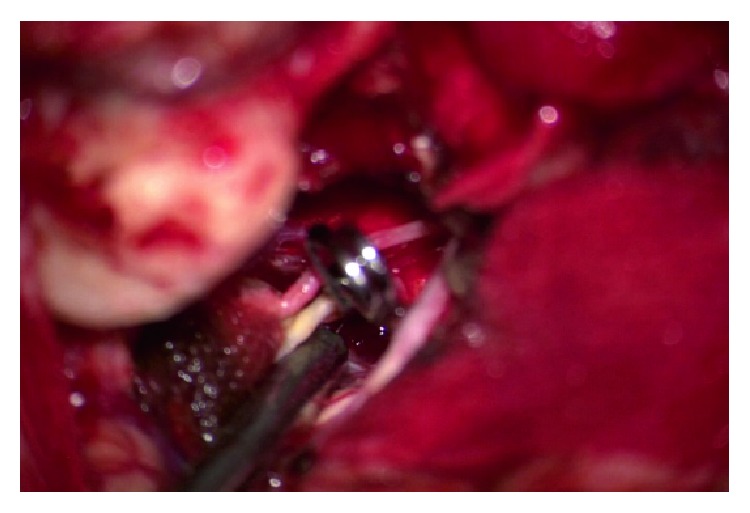
Clip applied.

**Figure 11:**
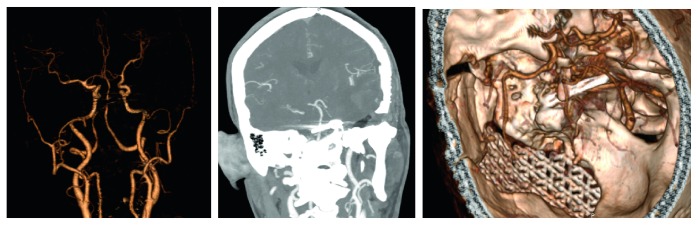
Post-op CTA.

The patient was extubated after 48 hours. Despite without motor deficit, CSF leak through the external auditory canal and facial paralysis (House-Brackmann Grade III) were observed. The management for the CSF leak was with lumbar drainage within 10 days. At three-month follow-up, the patient had full recovery of facial nerve palsy, and was able to walk normally. CTA showed that the aneurysm was totally occluded with no sign of basilar artery stenosis (Figures 7–11).

## 4. Discussion

### 4.1. Surgical Indication

In this endovascular era, surgical indication for the complex aneurysms becomes less and less. In 2004, the biggest series of operated AICA aneurysms were firstly reported by Gonzalez [7]. A Xialin' literature review (2016) through Pubmed reported 47 cases of anterior inferior cerebellar aneurysms (in 30 previous reports), in which 21 cases were treated surgically [8]. We did not find any other large series of AICA aneurysms operated in the literature. Most previous authors made case reports for only endovascular treatment of AICA aneurysms. For example, Kawase (1996) reported 14 cases of vertebrobasilar aneurysms, in which there were only 2 cases of AICA aneurysms [5]. In another publication, six cases of operated AICA aneurysms were reported by XinLi (2012) [9]. According to Gonzalez (2004), the endovascular coiling should be indicated for treating residual aneurysms after surgery, proximal aneurysms located anterior to the arteriovenous malformations, and high-grade patients with small aneurysms with an adequate dome-to-neck ratio. However, endovascular coiling has not been indicated for the large and giant aneurysms resulting in compression of the brainstem.

We did not choose the coiling treatment because of the mass effect on the brainstem. The radiologists performed the flow diversion for this case but they did not succeed. Finally, we decided the surgical clipping for giant AICA aneurysm using combined petrosectomy approach with two purposes: (i) eliminating the aneurysm, and (ii) relieving the mass effect on the brainstem.

### 4.2. Combined Transpetrosal Approach

Gonzalez (2004) performed hypothermic cardiac standstill in 9 patients with large and giant aneurysms. This surgical technique softens the aneurysmal sac, therefore, proximal control is no longer necessary [7]. At our hospital, we did not have the possibility for the cardiac standstill, so the approach for our patient must be large enough for both clipping and proximal control. The anterior petrosectomy is enough to clip AICA aneurysm. Inferior limit of anterior approach is internal auditory canal. AICA aneurysm surgery requires more space, especially for the proximal control. The posterior petrosectomy responded to this requirement.

In our case, the limitation of the combined transpetrosal approach was that its operating time was longer than others such as pterional, subtemporal, or pure Kawasé approach. It took us 5 hours 20 minutes for this approach, in which 3 hours 15 minutes is for the posterior petrosectomy. This long time for the posterior petrosectomy was recorded because some steps such as unroofing of the sigmoid sinus or skeletonizing the semicircular canal must be taken meticulously. However, we have not found any comment about the operating time concerning skullbase approaches in the literature.

Kawase was the first surgeon who used anterior petrosectomy for midbasilar aneurysm clipping. However, he realized that it was difficult to control the proximal artery [4]. Later, anterior petrosectomy was used for the tumoral lesions. Inferior limit of anterior approach was internal auditory canal. Aneurysm clipping required more space, especially for the proximal control in the large and giant aneurysms. Posterior petrosectomy responded to this requirement.

### 4.3. Clipping Technique

Based on general techniques, we put temporary clips on 2 vertebral arteries through the posterior petrosal opening in order to soften the giant aneurysm. The temporary clipping time was about 6 min. Calcification of aneurysmal wall, intraluminal thrombus can make this step challenging.

We had no difficulties in clipping this aneurysm because its projection was anterior-posterior and superior-inferior. Therefore, the clips could be put on the neck directly through the drilled Kawase triangle. However, because of the wide neck and thin wall, the aneurysm wall was torn during the first clip application. Fortunately, this first clip was put far from the real neck, hence, with the second clip, the laceration was completely closed. Lawton (2011) described the technique of “orientation”. He put the first long clip not too close to the aneurysmal neck, just to orientate the following clips [6, 8].

In this case, the anterior petrosectomy is sufficient enough for clip application. However, if the aneurysm is more complex or the projection is superiorly/anteriorly, maybe there will not be enough space for the manipulation. An extended approach to zygomatic bone may be necessary to gain more space for the surgeon.

### 4.4. Surgical Results

Giant AICA aneurysm was totally occluded. The mRankin was 1 after 3 months. In the earlier reports, good surgical outcome for the basilar aneurysms was about 80%, but no study was big enough to evaluate the surgical outcome of midbasilar aneurysm [5, 6], Mass effect disappeared after 3 months. This is also an advantage of clipping in comparison with endovascular treatments according to other authors' reports [8, 10].

## 5. Conclusion

Giant AICA aneurysm is a very rare lesion. Based on our experience in this case, we propose that combined transpetrosal approach is appropriate for surgical clipping. A good surgical outcome is achieved with the complete elimination of aneurysm.

## Data Availability

The data used to support the findings of this study are available from the corresponding author upon request.
